# Supporting public involvement in interview and other panels: a systematic review

**DOI:** 10.1111/hex.12491

**Published:** 2016-08-17

**Authors:** Susan Baxter, Mark Clowes, Delia Muir, Wendy Baird, Andrea Broadway‐Parkinson, Carole Bennett

**Affiliations:** ^1^ NIHR Research Design Service Yorkshire and Humber University of Sheffield Sheffield UK; ^2^ School of Health and Related Research University of Sheffield Sheffield UK; ^3^ NIHR Research Design Service Yorkshire and Humber University of Leeds Leeds UK; ^4^ Patient Involvement Member of NIHR RDS Yorkshire and Humber PPI Forum

**Keywords:** lay members, public involvement, public participation, public representatives, systematic review

## Abstract

**Background:**

Members of the public are increasingly being invited to become members of a variety of different panels and boards.

**Objective:**

This study aimed to systematically search the literature to identify studies relating to support or training provided to members of the public who are asked to be members of an interview panel.

**Search strategy:**

A systematic search for published and unpublished studies was carried out from June to September 2015. The search methods included electronic database searching, reference list screening, citation searching and scrutinizing online sources.

**Inclusion criteria:**

We included studies of any design including published and unpublished documents which outlined preparation or guidance relating to public participants who were members of interview panels or representatives on other types of panels or committees.

**Data synthesis:**

Results were synthesised via narrative methods.

**Main results:**

Thirty‐six documents were included in the review. Scrutiny of this literature highlighted ten areas which require consideration when including members of the public on interview panels: financial resources; clarity of role; role in the interview process; role in evaluation; training; orientation/induction; information needs; terminology; support; and other public representative needs such as timing, accessibility and support with information technology.

**Discussion and conclusions:**

The results of the review emphasize a range of elements that need to be fully considered when planning the involvement of public participants on interview panels. It highlights potential issues relating to the degree of involvement of public representatives in evaluating/grading decisions and the need for preparation and on‐going support.

## 1 INTRODUCTION

The expertize of members of the public has been described as a unique resource, which is completely different from professional skills.^1^ There is a growing literature reporting involvement of the public in the development of health‐specific interventions or to get feedback about a health‐care issues. Public involvement is increasingly moving beyond this level, with recognition of the need to engage citizens not only in providing feedback on health‐care delivery or interventions, but in processes whereby decisions are made.^2^ In healthcare, public involvement at all levels including Governing Boards has been advocated.^3^ Organizations such as INVOLVE have developed to support public participation in NHS, public health and social care research.^4^

Public representation on the majority of committees and panels is increasing, and the public are also often included on interview panels for health‐care staff and during the recruitment of prospective health‐care students.^5^ It is argued that lay people are in a good position to contribute to interview panels, in particular to evaluate communication skills, attitudes and values.^5^ In response to this growing involvement, there is a need for guidance to inform organizations planning involvement, or members of the public who have been asked to contribute.

The aim of this study was to search for and identify any research or guidance relating to members of the public taking part in interview panels. The work intended to use existing literature to underpin development of future guidance, to support members of the public who are asked to take part in interviewing.

## 2 METHODS

We carried out a rigorous and systematic review of published and unpublished literature relating to the involvement of members of the public in interview panels.

### 2.1 Identification of studies

#### 2.1.1 Search strategy

A systematic and comprehensive literature search of key health, medical, social services and business databases was developed by the information specialist on the review team (MC) and undertaken from June 2015 to September 2015. The searching process aimed to identify studies relating to the training of public representative members of interview panels or the impact or value of lay members being involved in interview panels. The search process was recorded in detail with lists of databases searched, date search run, limits applied, number of hits and duplication as per PRISMA guidelines.

An initial search was developed, comprising of terms relating to patient representation on interview panels. This initial search retrieved only a limited number of papers which specifically related to out topic area; patients on interview panels. When terms were added which related to training/guidance for patients to take part in interviews, very few citations remained. A second phase of searching was carried out in a wider set of databases, and using a broader range of terms. It took several iterations to get the search to a stage where it found a manageable number of citations, while still including studies that had been identified as relevant in the first search. Further details of the search strategy are provided as additional online material.

In addition to standard electronic database searching, citation searching was undertaken later in the project (September 2015) and reference lists of relevant papers were screened. Searches for UK grey literature were undertaken in order to identify any reports or evaluations of “grass roots” projects or other evidence not indexed in bibliographic databases, and also to minimize problems of publication bias. We searched websites of relevant patient and public involvement organizations such as INVOLVE and also extensively searched for online documents using Google, with identified sources and terms used in the titles of these documents used as terms for further searching.

#### 2.1.2 Sources searched

As it was anticipated that relevant literature would be dispersed across a number of disciplinary fields, a wide variety of data sources were searched including: MEDLINE, EMBASE, PsycINFO and Social Policy & Practice (via Ovid); CINAHL, BNI, HMIC, Health Business Elite (via the HDAS platform on the NICE website) and Social Care Online (via the SCIE website). Simpler, high‐sensitivity searches were conducted of ASSIA, IBSS and Social Services Abstracts (via ProQuest), from which additional results were selected by hand. Searches were limited to the year 1995 onwards, encompassing 20 years of research. All citations retrieved by the searching were imported into EndNote, and duplicates were deleted prior to scrutiny.

#### 2.1.3 Search restrictions

We included work published in developed countries ([members of the Organisation for Economic Collaboration and Development [OECD]) and published in English or with an English abstract.

### 2.2 Selection of papers

#### 2.2.1 Inclusion and exclusion criteria

##### Population

We aimed to include studies that related to public participation in interviews. A variety of terms may be used to describe this population including: lay member; patient and public representative; patient representative; expert patients; service users; and community members, consumers and lay advisors. Rather than attempting to define who we would consider to be “the public,” we defined by exclusion, so considered any studies that related to individuals who were *not* professionals or *not* staff members employed by the organization hosting the panel. We searched for public involvement taking place in any context, including business, industry, public and private organizations. As the terms “participation” and “involvement” can encompass a wide variety of activities and levels of inclusion, we deliberately adopted a broad definition. We searched for literature which described public individuals having any role in interviews or membership of a committee or panel, in a health‐care context.

##### Interventions

We searched for studies which outlined preparation or guidance relating to public participants who were members of interview panels. Due to the limited body of literature we located, we widened our criteria from interview panels to include public representatives on other types of panels or committees. The review focused on the process whereby the public was involved in decision‐making activities, rather than looking for documents where public input had been sought (such as reports or guideline development). We scrutinized any literature describing involvement in interviews/panels/committees, looking for elements relating to preparation and training of public representatives.

##### Outcomes

We searched for literature which reported any outcomes following public representatives taking part in interviews or other panels. These could include actual or perceived benefits of members of the public taking part. We also included literature which did not report outcomes, and instead described the experiences of members of the panel, or provided guidance or policy recommendations.

##### Study design

Any study designs were eligible for inclusion, with articles that were more descriptive, in addition to empirical studies which reported data/results.

### 2.3 Process of selection of studies

Citations retrieved via the searching process were uploaded to an EndNote database. This database of study titles and abstracts was screened by SB and MC. Full documents of all citations coded as potentially relevant were then retrieved for systematic screening.

### 2.4 Data extraction strategy

Studies which met the inclusion criteria following the selection process above were read in detail and data extracted. An extraction form was developed to ensure consistency in data retrieved from each study.

### 2.5 Quality appraisal strategy

We carried out quality evaluation only for the journal articles. We used the Centre for Evidence‐based Medicine tool for critical appraisal of surveys.^6^ This instrument requires a yes/no/can't tell response to 12 questions. For cohort (before and after) and mixed method studies, we used items from the Critical Skills Appraisal Tools checklist^7^ with a yes/no/can't tell response to eight questions. For the qualitative/action research studies, we used the CASP qualitative checklist 10 questions.^8^ The full tools used are available as additional online material.

## 3 RESULTS

Our searches identified 36 documents/papers that contained information relating to support, training or guidance for public representative members of interview panels, other panels and committees, or boards that had public members.

### 3.1 Quantity of the literature available

**Figure 1:**
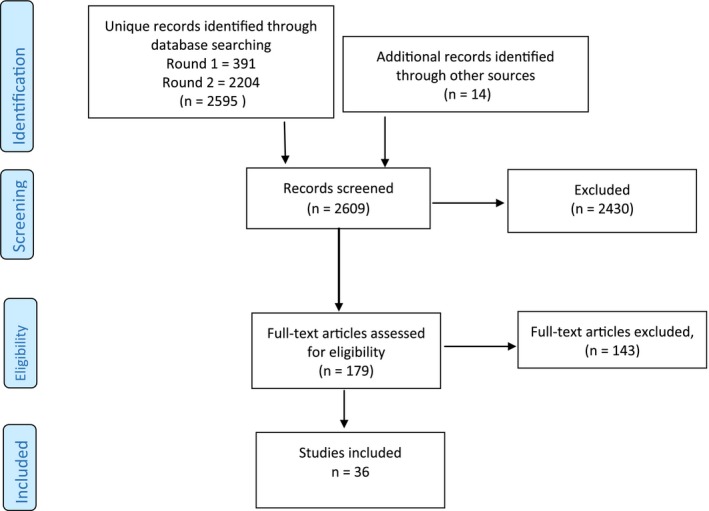
The selection process

Figure 1 provides a detailed illustration of the process of study selection which led to the inclusion of the 36 documents. The initial electronic database searches using terms related to patient involvement in interviews identified 391 citations following de‐duplication. From this database of citations, 59 potentially relevant papers were retrieved for further scrutiny, and of these, five met the criteria for inclusion.

The second electronic database search using broader terms in more databases identified an additional 2204 citations that had not already been retrieved. From these, 33 potentially relevant papers were retrieved for further scrutiny. Examination of these articles resulted in an additional 21 papers that met the inclusion criteria for the review. In addition to these papers which were identified from electronic database searching, ten further documents were included from additional searching strategies (citation searching, reference list scrutiny or web searching).

### 3.2 Type of the literature available

Our target literature related to public representation on interview panels, and we identified eight documents that related to this (see Table 1).^5^, ^9^, ^10^, ^11^, ^12^, ^13^, ^14^, ^15^ One additional paper^16^ described involvement in preparing interview questions, although not in the interview sessions. A further six documents considered public membership of other panels, such as those dealing with clinical audit, complaints or accreditation.^17^, ^18^, ^19^, ^20^, ^21^, ^22^ We identified seven documents that related to public membership of committees, such as research ethics committees.^23^, ^24^, ^25^, ^26^, ^27^, ^28^, ^29^ The other papers we included related to public membership of boards (11 documents)^2^, ^30^, ^31^, ^32^, ^33^, ^34^, ^35^, ^36^, ^37^, ^38^, ^39^ or relevant documents outlining training programmes for enhancing public involvement more generally (three papers).^1^, ^40^, ^41^

**Table 1:** Literature categorized by role of public representative

Role of public representative	Included studies
Public members of an interview panel	Browne et al. (2015)^9^
	Burket et al. (2005)^10^
	Department of Health (2004)^11^
	Hurtado et al. (2012)^12^
	Matka et al. (2010)^13^
	Roberts et al. (2010)^5^
	Richardson et al. (2013)^14^
	University of Keele (2015)^15^
Involvement in interview process	Anghel & Ramon (2009)^16^
Public members of other panels	Healthcare Quality Improvement Partnership (2013)^17^
	Jones & Royse (2008)^18^
	Monahan & Stewart (2003)^19^
	NHS Wales (2003)^20^
	O'Connor et al. (2007)^21^
	Oliver (2001)^22^
Public members of committees	Buckland et al. (2009)^23^
	Department of Health (2006)^24^
	Fletcher & Buggins (1997)^25^
	Gilbert (2012)^26^
	NHS West Sussex (2010)^27^
	O'Hara et al. (2015)^28^
	Ukpong (2012)^29^
Public members of boards	Dougherty & Easton (2011)^30^
	Frankish (2002)^31^
	Greco (2004)^32^
	Health and Care Professions Council (2013)^33^
	Jenkinson et al. (2014)^34^
	Jennings & Smith (2015)^35^
	Klitzman (2012)^36^
	NHS England (2015)^37^
	Oxfordshire (2010)^38^
	Pickard (2001)^2^
	Wandsworth CCG (2013)^39^
Training for public involvement roles	Lockey et al. (2004)^40^
	Mosconi et al. (2012)^1^
	Parkes et al. (2014)^41^

Table 2 provides a summary of the types of documents in the included set. As can be seen, just over half (19) of the documents we included were papers published in peer‐reviewed journals. We identified a small number of reports (four), and 13 online documents, which ranged from one‐ or two‐page guidance/recommendations to more substantial training manuals or guidelines.

**Table 2:** Literature categorized by type of document

Document type	Included studies
Journal article	Anghel & Ramon (2009)^16^
	Browne et al. (2015)^9^
	Burket et al. (2005)^10^
	Dougherty & Easton (2011)^30^
	Frankish (2002)^31^
	Greco (2004)^32^
	Hurtado et al. (2012)^12^
	Jenkinson et al. (2014)^34^
	Jones & Royse (2008)^18^
	Klitzman (2012)^36^
	Matka et al. (2010)^13^
	Monahan & Stewart (2003)^19^
	Mosconi et al. (2012)^1^
	O'Connor et al. (2007)^21^
	Oliver (2001)^22^
	Parkes et al. (2014)^41^
	Pickard (2001)^2^
	Richardson et al. (2013)^14^
	Roberts et al. (2010)^5^
Report	Healthcare Quality Improvement Partnership (2013)^17^
	Lockey et al. (2004)^40^
	Fletcher & Buggins (1997)^25^
	O'Hara et al. (2015)^28^
	Buckland et al. (2009)^23^
Online document	Department of Health (2004)^11^
	Department of Health (2006)^24^
	Gilbert (2012)^26^
	Health and Care Professions Council (2013)^33^
	Jennings & Smith (2015)^35^
	NHS England (2015)^37^
	NHS West Sussex (2010)^27^
	NHS Wales (2003)^20^
	Oxfordshire (2010)^38^
	Ukpong (2012)^29^
	University of Keele (2015)^15^
	Wandsworth CCG (2013)^39^

The literature was dominated by studies originating from the UK (24 documents). This may be in part attributable to the inclusion of UK unpublished literature. Six articles originated from the United States and two from Australia (see Table 3).

**Table 3:** Literature by country of origin

Country	Included studies
UK	Anghel & Ramon (2009)^16^
	Burket et al. (2005)^10^
	Department of Health (2004)^11^
	Department of Health (2006)^24^
	Gilbert (2012)^26^
	Greco (2004)^32^
	Health and Care Professions Council (2013)
	Healthcare Quality Improvement Partnership (2013)^17^
	Hurtado et al. (2012)^12^
	Jennings & Smith (2015)^35^
	Lockey et al. (2004)^40^
	Matka et al. (2010)^13^
	Fletcher & Buggins (1997)^25^
	NHS England (2015)^37^
	NHS West Sussex (2010)^27^
	NHS Wales (2003)^20^
	Oliver (2001)^22^
	Oxfordshire (2010)^38^
	Parkes et al. (2014)^41^
	Pickard (2001)^2^
	Richardson et al. (2013)^14^
	Roberts et al. (2010)^5^
	Buckland et al. (2009)^23^
	University of Keele (2015)^15^
	Wandsworth CCG (2013)^39^
USA/Canada	Dougherty & Easton (2011)^30^
	Frankish (2002)^31^
	Jones & Royse (2008)^18^
	Klitzman (2012)^36^
	Monahan & Stewart (2003)^19^
	O'Hara et al. (2015)^28^
Australia	Browne et al. (2015)^9^
	Jenkinson et al. (2014)^34^
Ireland	O'Connor et al. (2007)^21^
Italy	Mosconi et al. (2012)^1^
Nigeria	Ukpong (2012)^29^

#### 3.2.1 Quality of the literature

The included studies generally used less robust designs (see Table 4), with only one paper^29^ including any form of comparator group, and two studies including initial (baseline/before) data, which were then compared to data collected later (follow‐up/after).^32^, ^34^ The most common approach used by researchers to evaluate public involvement was self‐reported surveys (questionnaires) that were completed by those taking part in an event.

**Table 4:** Literature by research design

Type of study design	Included studies
Controlled before and after	Ukpong (2012)^29^
Before and after	Greco (2004)^32^
	Jenkinson et al. (2014)^34^
Mixed method	Anghel & Ramon (2009)^16^
	O'Connor et al. (2007)^21^
Qualitative/action research	Klitzman (2012)^36^
	Lockey et al. (2004)^40^
	Oliver (2001)^22^
Survey (questionnaire)	Burket et al. (2005)^10^
	Dougherty & Easton (2011)^30^
	Jones & Royse (2008)^18^
	Matka et al. (2010)^13^
	Monahan & Stewart (2003)^19^
	Mosconi et al. (2012)^1^
	O'Hara et al. (2015)^28^
	Parkes et al. (2014)^41^
	Pickard (2001)^2^
	Roberts et al. (2010)^5^
	Buckland et al. (2009)^23^
Descriptive	Browne et al. (2015)^9^
	Frankish (2002)^31^
	Gilbert (2012)^26^
	Hurtado et al. (2012)^12^
	Fletcher & Buggins (1997)^25^
	NHS Wales (2003)^20^
	Richardson et al. (2013)^14^
Policy document/guidance	Department of Health (2004)^11^
	Department of Health (2006)^24^
	Health and Care Professions Council (2013)^33^
	Healthcare Quality Improvement Partnership (2013)^17^
	Jennings & Smith (2015)^35^
	NHS England (2015)^37^
	NHS West Sussex (2010)^27^
	Oxfordshire (2010)^38^
	University of Keele (2015)^15^
	Wandsworth CCG (2013)^39^

The quality of the survey studies was affected by few of them using tools which were described as being previously validated, tested or piloted. The achievement of a reasonable response rate to surveys is always challenging, and some of these studies reported <50% response. For some of the studies, the authors reported only the number of completed questionnaires, and not the figure for those distributed. The group of survey studies predominantly reported findings as percentages, with few using statistical tests. The papers of other study designs were frequently limited by unclear/lack of reporting of elements, and limited reporting of study findings. See Table 5 for completed quality appraisals.

**Table 5:** Quality appraisal of the included studies

Study design	Authors	Elements of quality assessed										
Survey		Focused question?	Design appropriate?	Selection method/bias?	Sample representative?	Based on sample size calculation?	Response rate satisfactory?	Questionnaire valid/reliable?	Statistical significance test used?	Confidence intervals?	Confounding factors?	Applicable?
	Burket et al. (2005)^10^	Y	Y	N	Y	N	Y	U	Y	N	N	Y
	Dougherty & Easton (2011)^30^	Y	Y	N	N	N	N	U	N	N	N	Y
	Jones & Royse (2008)^18^	Y	Y	U	Y	N	U	U	N	N	U	Y
	Matka et al. (2010)^13^	Y	Y	N	U	N	U	U	N	N	U	Y
	Monahan & Stewart (2003)^19^	Y	Y	U	U	U	N	U	Y	N	N	Y
	Mosconi et al. (2012)^1^	Y	Y	N	N	N	Y	U	N	N	N	Y
	O'Hara et al. (2015)^28^	Y	Y	N	U	N	Y	U	N	N	U	Y
	Parkes et al. (2014)^41^	Y	Y	N	U	N	U	U	N	N	U	Y
	Pickard (2001)^2^	Y	Y	N	Y	N	Y	Y	N	N	U	Y
	Richardson et al. (2013)^14^											
	Roberts et al. (2010)^5^	Y	Y	N	Y	N	U	U	N	N	N	Y
	Buckland et al. (2009)^23^	Y	Y	N	N	N	N	U	N	N	N	Y
Before and after		Focused issue?	Recruitment acceptable?	Exposure bias?	Outcome bias?	Confounding factors?	Follow‐up complete?	Follow‐up length adequate?	Reporting is precise?	Results are believable?		
	Greco (2004)^32^	Y	U	N	N	U	Y	N	Y	Y		
	Jenkinson et al. (2014)^34^	Y	Y	N	Y	U	N	Y	Y	Y		
	Ukpong (2012)^29^	Y	U	U	U	U	U	U	U	Y		
Mixed method	Anghel & Ramon (2009)^16^	Y	U	U	N	N	n/a	n/a	N	Y		
	O'Connor et al. (2007)^21^	Y	N	N	N	N	n/a	n/a	Y	Y		
Qualitative/action research		Clear aims?	Methodology appropriate?	Design appropriate?	Recruitment strategy appropriate?	Data collection issues?	Researcher relationship considered?	Ethical issues taken into account?	Data analysis rigorous?	Clear statement of findings?	Research valuable?	
	Klitzman (2012)^36^	Y	Y	Y	Y	Y	N	N	Y	Y	Y	
	Lockey et al. (2004)^40^	Y	Y	Y	Y	Y	N	Y	Y	Y	Y	
	Oliver (2001)^22^	Y	Y	Y	Y	Y	N	N	Y	Y	Y	

#### 3.2.2 Narrative summary of the literature

Analysis of the included documents identified a range of elements that need to be fully considered when involving the public in interviewing and other types of panels. These elements will now be summarized. Full extraction tables for the included studies are available as additional online material.

##### Financial resources

The first aspect requiring consideration that was described in the studies, related to resourcing public involvement in interviewing, remuneration and the employment status of public members.

The authors of a study evaluating the involvement of service users in a social work training programme^16^ described how they had intended to have users as co‐interviewers, but had lacked the financial resources to pay them. This had led to their involvement being limited to suggesting interview questions. In another included paper, Gilbert^26^ emphasized that financial or other recognition must be agreed in advance of implementing public involvement in interviews, with arrangements made for reimbursing travel costs or covering care. Roberts et al.^5^ and Richardson et al.^14^ similarly highlighted that resources need to be allocated. In the first of these^5^ which evaluated the inclusion of lay people in student nurse interviews, the “lay involvement assistants” were employed on casual contracts.

A report^17^ providing guidance on developing patient panels describes the issuing of honorary contracts. An example of information provided to lay council members also included details of remuneration.^33^ In another study, people with learning difficulties who were involved in recruiting staff received £50 vouchers to recognize their contribution.^12^ In contrast, a document providing information for lay interviewers of prospective medical students^15^ describes the work as voluntary and unpaid, with reimbursement only of travelling expenses.

In a Canadian survey of lay representatives on health research committees,^28^ around half were reportedly reimbursed in the form of a payment, meal, travel, conference attendance or general expenses. In another study^23^ exploring views regarding lay members of research ethics committees, responses were evenly spread in regard to whether they should be paid an allowance for their work. A qualitative study of training for involvement in research^40^ highlighted the need to fully consider the issue of whether service users should be paid to undertake any training necessary for their role.

##### Clarity of role

The most commonly described element requiring consideration was ensuring clarity of the role. Several of the documents highlighted the need for or provided examples of role descriptions as part of information provided to potential participants. One example of online guidance^39^ described a need to be clear regarding what is expected of the public participant and what the boundaries are. It emphasized that a role or job description is needed, which should set out key tasks that will be required. A document providing information for prospective interviewers^15^ similarly detailed what the role would be and how the individual would be contributing to the process. It also provided a list of required attributes in the form of a “skills profile.” The Roberts et al.^5^ study of involvement in nurse student interviews described the development of a role description and person specification. Other included documents^24^, ^26^, ^27^, ^33^, ^35^, ^38^ also emphasized the need for role descriptions and clarity regarding roles and expectations.

Some of the included literature described issues with clarity of roles and responsibilities. Pickard^2^ found in a UK survey study that the role of lay members on Primary Care Boards was ill‐defined, and as a result, there was variation between organizations in how involved lay members were in actual decision making.

A Canadian paper^31^ discussed the challenges associated with citizen participation in Regional Health Boards/Councils. This study reported differing opinions among board members regarding appropriate roles and responsibilities of lay members and the capacity of citizen participants to make health system decisions. A second paper from Canada^19^ also found that professional and lay member perceptions of lay involvement in Grant Review Panels could differ. Another non‐UK study^36^ echoed these findings, describing confusion regarding role for lay members of University Research Panel members, with members appearing to have widely varying roles and functions that they were asked to fulfil.

In contrast to these studies reporting variation in perceptions of roles, another paper^28^ found that there was a high degree of agreement between the way non‐lay representatives on health research committees saw their lay colleagues and the way lay representatives saw themselves. This work, carried out a decade after the earlier two Canadian papers, may indicate that a shift in perceptions had taken place over this time.

A study of public involvement in accreditation assessments^21^ described how some service user members provided only a verbal input to the report, whereas others contributed a written input. All participants in the evaluation of the system reported that there was a role for user members in feeding back/debriefing. This had led to the process being changed, with public members provided with a template for written reports, or given the option of presenting feedback verbally.

Another important element to consider regarding role was highlighted in four documents. These discussed who the public member was considered to be representing. A policy document^27^ outlined that lay members are not expected to represent the views of the wider community (unless recruited because they are a member of a specific group and are authorized to speak on behalf of that group). O'Hara et al.^28^ emphasized that the question of what community does the representative serve is important. Pickard^2^ reported that there was some ambiguity around who the lay members of Primary Care Boards believed themselves to be representing. In similar vein, an investigation of community members of institutional review boards found that there was confusion regarding who the members were representative of.^36^

##### The interview process

Five papers highlighted the need to consider how public participants would take part in the interview process. The service users in one study^16^ suggested questions, but did not take part in the interviews. Matka et al.^13^ in contrast outlined that a service user and a clinician interviewed each applicant together. Each interviewer evaluated the candidates’ response using predefined model answers, and interpersonal skills were also assessed by both. Richardson et al.^14^ described panels which included lay representatives, with all members contributing to asking questions. An alternative model described was of lay assistants meeting the applicants separately for half an hour before the interview and not taking part in student interviews.^5^ Lay members were given tasks to do with the candidates around personal qualities and specific questions. Another study^12^ similarly described a two‐panel process (a professional interview panel and a service user panel), with each focusing on a different aspect of the person specification. The user panel assessed aspects of behaviour and character using a pre‐established scoring criteria. The professional panel evaluated skills and knowledge. Both panels joined at the end of the day to collaboratively make a decision.

##### Role in evaluation

A particular area which reportedly required consideration was the role of the public representative in evaluation or scoring. Gilbert^26^ advised that a clear rationale should be present for the voting/assessment strategy employed. Matka et al.^13^ reported that there were some tensions about user capacity to assess applicants to a social work programme, with a need for academics to take the final decision and have the final responsibility regarding the outcome of the interview. In a study examining interviewing procedures for new staff,^12^ the professional panel scoring had a higher weighting than the user panel. However, if the user panel had significant concerns, a candidate would not be offered the post. The authors recommended that there should be a pre‐agreed plan in the event of a significant disagreement. An examination of involvement in nurse interviews^5^ reported that the lay assessment did not affect the outcome of the interview (but this was intended to be changed in the future). The authors emphasized the need to have strategies in place to deal with any differences of judgement. Another study of prospective student interviews^14^ described initial concerns regarding differing opinions between panel members. However, they found that there was usually agreement.

O'Connor et al.^21^ described a number of issues in relation to the role of the service user assessors in the accreditation assessments of health‐care organizations. Some were asked to contribute to the rating of the organization and others were not, depending on who the team leader was. The authors reported that following their evaluation, this has now been clarified and staff facilitate the contribution of users to rating. In support of users having full involvement in evaluation, a paper^10^ reported that overall comparison of scores given to candidates applying for GP training revealed no significant difference between the marks awarded by medical and lay assessors (*P*=.79). In this study, all assessors had attended the same training day which included discussion of scoring using video examples.

##### Training

The included documents emphasize the importance of training for public representatives before taking part in panels. Roberts et al.,^5^ for example, asserted that lay people should be briefed fully and initial and on‐going training should be provided. Another study^28^ reported that education enables well‐informed and productive lay representatives. The researchers in this survey suggested that education needs to be on‐going, with training coming from multiple sources—online, face‐to‐face mentoring, workshops and other forms of instruction. On‐going training was similarly recommended by Ukpong^29^ with update training at least once a year.

Three documents described a lack of training for patient representatives. Dougherty and Easton^30^ found that lay board members usually received no training or orientation. Variability in practice was described in a study of research review boards.^36^ A few had a full‐time staff member to orient and train lay individuals, for others public members were told “just do what everyone else is doing.” Similarly, another study^2^ found that training was sporadic and varied considerably.

#### 3.2.3 Elements of training for involvement

Two papers described the benefits of the same training being provided to both lay and professional panel members.^10^, ^24^ Training was recommended in the following areas:

1. Knowledge of equal opportunities/equality and diversity.^11^, ^14^, ^15^
2. Recruitment processes.^11^, ^14^
3. Confidentiality and data protection.^9^, ^11^, ^14^, ^26^ Three organizations reported that they required a confidentiality agreement to be signed.^17^, ^27^, ^39^ In addition, the need to have a Criminal Records Bureau check was commonly described.
4. The role of lay members.^5^, ^14^, ^20^, ^21^, ^41^
5. Effective communication and personal attributes such as confidence; influencing decision making; developing the skills of dialogue; conveying the views of others; asking for clarification; commenting; challenging health professionals when necessary; putting ones views across.^16^, ^18^, ^22^, ^25^, ^26^, ^32^, ^34^

Several studies described the importance of tailoring training to the individual needs of public representatives. Gilbert,^26^ for example, recommended that training should be co‐produced with lay members, with an emphasis on what is important to them. Lockey et al.^40^ reported that training was most valued when it had a clear purpose and was centred on specific tasks or real problems. Participants valued trainers who facilitated the interaction and exchange of ideas, and reported that training should be in a safe environment, with time and space to allow contributions. Some respondents in the O'Hara et al.^28^ study reported that they had sufficient expertize to underpin their role as lay representatives on health research committees and as such did not require additional training.

##### Orientation/induction

Several documents described the importance of orientation/induction meetings. Roberts et al.,^5^ for example, provided an initial briefing session consisting of an introduction to the school, introduction to the project, a tour of the facilities, a chance to contribute ideas and ask questions. There was also an informal lunch for everyone that would be involved in the interviews to meet each other. The Healthcare Quality Improvement Partnership^17^ similarly recommends a chance for public representatives to meet the team. A training programme for lay members of panels^20^ recommended that participants sit in on a session beforehand. O'Connor et al.^21^ reported that service user members felt training could be improved by introducing a shadowing component. One paper^14^ described the induction process as being important in order to build relationships with the service users and for the service users to highlight any concerns or identify special requirements.

##### Information needs

Included documents described the need for public representatives to be provided with a range of information. One^39^ detailed that representatives will need help in understanding the structure and working relationships of the NHS and that information or training on this should be provided. Another document^37^ suggested that an information pack provided to potential lay members should include: introduction about the organization; details of time commitment, remuneration and induction if appointed; disqualification criteria; and governance/public life standards. Other documents reported the need to provide information on:

1. National and local services, and description of the terms of reference of the committee^24^
2. Information about the council and its role together with the aim of recruiting lay members^33^
3. The rationale for involvement in admissions;^13^
4. Understanding how the NHS works, standards and complaints procedure;^20^
5. The steps in the process including timings and a sheet providing examples of question types that were to be used in the interviews.^5^
6. Meeting schedules, contact details, historical and local context, the role of the board and lay members.^38^

##### Terminology

Language was often mentioned as a barrier to meaningful involvement in panels.^23^, ^40^ Specialist jargon could be alienating, but business was described by committee members as being impeded if everything was “translated” into non‐specialist language.^22^ While it was highlighted that unnecessary terminology should be avoided,^26^ there was a focus on lay members needing to learn terminology in order to be included. It was suggested that public participants should receive a glossary of terms or “jargon buster”^22^, ^27^, ^35^, ^38^ or be trained in terminology.^32^, ^40^

##### Support

Five documents described the importance of support for public participants. Two^27^, ^28^ recommended that there should be at least two members to provide each other with support and that public representatives should have an identified team member contact details. Similarly, another document^24^ suggested pairing with a buddy and handover between lay members. The induction process for a Safeguarding Children Board included a buddy system with new members paired up with those more experienced.^38^ Oliver^22^ reported that learning from others was a recurring theme; new panel members learnt from more experienced colleagues. New members in this study appreciated an induction day, but found this only a beginning to a long apprenticeship, and described the value of on‐going support via mentorship. Support suggested for new members of panels included: practice sessions; someone to phone when something was unclear; and opportunities to meet others.

##### Consideration of other public member needs

Other elements highlighted as contributing to successful public involvement included ensuring that the venue was accessible,^24^, ^27^ sufficiently heated^14^ and also that the timing of sessions was convenient.^24^, ^26^ Two studies described the need to consider Internet access and information technology support.^17^, ^26^

Another aspect of timing related to the time constraints and commitment of public members. Documents detailed the need to send papers well ahead of a meeting.^26^ Buckland et al.^23^ reported that factors hindering involvement included the amount of paperwork involved, the amount that needed to be covered in meetings and the accessibility and timing of meetings and training.

## 4 DISCUSSION

This study aimed to systematically identify and summarize research or guidance relating to members of the public taking part in interview panels. We found only seven documents which specifically related to interview panels. Of these, four described services user/lay people taking part in selection panels for prospective health‐care professional trainees and three outlined service user/lay members on staff recruitment panels. Other documents which we included related to public membership of other forms of panels or committees that were relevant to interview panels. Scrutiny of these two different groups of documents (interview panels vs other panels and committees) indicated common issues across them and supported our decision to extend the review to a broader inclusion criteria.

The body of literature was supportive of including public participants on panels, and several studies described benefits such as adding credibility and balance to the process. There were some reports of disquiet among professional panel members in regard to the role of public members. This highlights the need for negotiating roles and establishing clear expectations regarding the role of public members in the process.

In some of the studies, public participants did not have an equal value in evaluation/scoring or only rated particular areas such as interpersonal skills. This raised questions regarding the value that is being attached to the contribution of the public member. One of the included papers^2^ described the lay member role in decision making as being only peripheral, highlighting a need to consider what true involvement in the process is. There may be particular circumstances in which involvement in assessment is not possible, and the rationale for this should be carefully considered. Several studies cautioned for a need to develop a strategy for situations where there was differing opinion among panel members.

This review of the literature highlighted the need for adequate planning and preparation prior to involvement of public members on panels. The literature described the need for orientation or induction sessions such as informal visits, sitting in on sessions and opportunities to meet fellow interview panel members. Studies highlighted that public panel members should receive a range of information in advance of a session, which may include explanations of relevant terminology. While the literature had a focus on members needing to learn terminology, better practice would be to ensure that sessions are conducted using language that everybody understands.^42^

Five of the included studies evaluated training that had been provided to public participants (maternity service users, non‐executive NHS directors, ethics committee members, patient organizations and child protection panels). These reported a positive impact on knowledge, and also on perceived confidence, and perceived effectiveness of public panel members. The literature highlighted that training enabled public representatives to be fully engaged and effective panel members. More informal support and preparation methods were also valuable, for example mentoring. The range of approaches seen in these papers demonstrates the importance of basing preparation and support around the needs and existing capacities of the individuals. This may differ according to the varying roles required in different organizations. The included studies predominantly related to longer term involvement for individuals. Where public members are invited for on–off sessions, their preparation needs may be different.

This systematic review of the literature endeavoured to be comprehensive and inclusive in its search for relevant documents. However, the literature used a wide variety of terms to refer to public participants. This made searching more challenging and raises the possibility of relevant work not being retrieved in our searches. We identified and examined a large body of online material, although only scrutinized the first few pages of returned results due to the range of search terms which were required.

The body of literature that we found was of limited quality, with a predominance of descriptive studies or those using a self‐evaluated survey design. We echo the findings of other reviews of public involvement^43^ which called for more research in the area and for the robust examination of public involvement in order that strategies may be evidence‐based.

## 5 CONCLUSIONS

The review indicates that public membership on interview panels was considered valuable, although a range of factors need to be fully considered when planning involvement. In particular, the intended role of the public member on the panel requires attention, together with sufficient preparation prior to the session and tailored on‐going support.

## CONFLICTS OF INTERESTS

No conflict of interests are declared.

## SOURCE OF FUNDING

The Research Design Service Yorkshire and Humber is funded by the National Institute for Health Research. The views and opinions expressed are those of the authors and do not necessarily reflect those of the NIHR, NHS or the Department of Health.
