# Supplementary material for: Supporting public involvement in interview and other panels: a systematic review
Source: Health Expect. 2016 Aug 17;20(5):807–17. doi: 10.1111/hex.12491 (PMC5600233; doi:10.1111/hex.12491)
Supplement: Supplementary file 1 [file HEX-20-807-s001.docx]

**ADDITIONAL ONLINE MATERIAL**

**Search strategy**

Example search strategy for Medline via Ovid (similar strategies were also run on EMBASE and PsycINFO).

| 1 | *Patient Participation/ |
| --- | --- |
| 2 | *Consumer Participation/ |
| 3 | *Advisory Committees/ |
| 4 | (train* or prepar* or guideline* or guidance or briefing* or briefed or skill* or role or (provid* adj3 information)).ti,ab. |
| 5 | (panel* or committee* or board* or jury or juries).ti,ab. |
| 6 | ((communit* or consumer* or client* or citizen* or user* or carer* or patient* or nonpatient* or public or communit* or lay or disabled) adj1 (participat* or involv* or member* or join* or invit* or representat* or partnership* or engag*)).ti,ab. |
| 7 | 1 or 2 or 6 |
| 8 | 3 or 5 |
| 9 | 4 and 7 and 8 |
| 10 | limit 9 to english language |
| 11 | limit 10 to yr="1995 -Current" |

**Quality appraisal tools**

**Tool for appraising surveys**

1. Did the study address a clearly focused question/issue?

2. Is the research method (study design) appropriate for answering the research question?

3. Is the method of selection of the subjects (employees, teams, divisions, organizations) clearly described?

4. Could the way the sample was obtained introduce (selection) bias?

5. Was the sample of subjects representative with regard to the population to which the findings will be referred?

6. Was the sample size based on pre-study considerations of statistical power?

7. Was a satisfactory response rate achieved?

8. Are the measurements (questionnaires) likely to be valid and reliable?

9. Was the statistical significance assessed?

10. Are confidence intervals given for the main results?

11. Could there be confounding factors that haven’t been accounted for?

12. Can the results be applied to your organization?

**Tool for appraising cohort/before and after studies**

1. Did the study address a clearly focused issue?

2. Was the cohort recruited in an acceptable way?

3. Was the exposure accurately measured to minimise bias?

4. Was the outcome accurately measured to minimise bias?

5. Have the authors identified and taken account of all important confounding factors?

6. Was the follow up of subjects complete and long enough?

7. How precisely are the results reported?

8. Are the results believable?

**Tool for appraising qualitative studies**

1. Was there a clear statement of the aim of the research?

2. Is a qualitative methodology appropriate?

3. Was the research design appropriate to address the aims of the research?

4. Was the recruitment strategy appropriate to the aims of the research?

5. Were the data collected in a way that addressed the research issue?

6. Has the relationship between researcher and participant been adequately considered?

7. Have ethical issues been taken into account?

8. Was the data analysis sufficiently rigorous?

9. Is there a clear statement of findings?

10. How valuable is the research?

**Extraction tables**

| Reference  1st author year | Anghel & Ramon, 2009 |
| --- | --- |
| Type of study/document | Journal article |
| Study design | Action research, survey and interviews |
| Country | UK |
| Participants/intended audience | 18 Service users described as “consultants”, together with staff delivering the programme |
| Research question/objectives | To describe and evaluate the involvement of service users in a social work training programme |
| Research methods | A mixture of qualitative and quantitative methods. |
| Results/data | Service users and carers were “partially involved” in the admission process by suggesting questions included within the admission interview. The team looked at having users as co-interviewers but lack the financial resources to pay them to be present for the more than 250 interviews.  The evaluation feedback resulted in training offered to users by site co-ordinators. The training focused on gaining confidence, effective presentation skills, dealing with disclosure and any issues.  Issues with changing the culture, and in having limited time and funding. |
| Main findings | Involvement in interviews requires funding. |
| Comments |  |

| Reference  1st author year | Browne & Lakeman, 2015 |
| --- | --- |
| Type of study/document | Journal letter to the Editor |
| Study design | Descriptive |
| Country | Australia |
| Participants/intended audience | Researchers |
| Research question/objectives | To describe issues around having service users on interview panels in mental health |
| Research methods | Description only |
| Results/data | The experience of the authors is that service user panellists apply themselves diligently, impartially and collegially. They suggest that at least one service users should be on the interview panel for any and all positions in a mental health service. The authors describe a suggestion that service users should be out of an area as they may hold a grudge or be admitted to an inpatient unit afterwards. They refute this as an issue and argue that having users on panels gives them a voice in important decision-making. |
| Main findings | Raises a potential issue of service users having knowledge/links to interviewees |
| Comments |  |

| Reference  1st author year | Buckland et al. 2009 |
| --- | --- |
| Type of study/document | Report |
| Study design | Survey |
| Country | UK |
| Participants/intended audience | 225 Lay members of research ethics committees |
| Research question/objectives | To describe lay member perspectives and involvement in RECs |
| Research methods | Members received a letter asking them to complete an online survey or return the questionnaire by post |
| Results/data | 92% of members reported that their views were taken into account. A large proportion of members rated the training they had received as helpful. Responses were evenly spread in regard to whether lay members should be paid an allowance for their work (38% yes, 30% no, 32% not sure). The main barrier identified was the language used by researchers. Other factors that could hinder involvement were the amount of paperwork involved, the amount that needed to be covered in meetings, accessibility and timing of meetings and training, lack of induction training, lack of clarity on legal issues, lack of understanding of technical issues, poor chairing, a lack of support from other members. |
| Main findings | Describes factors impacting on involvement. |
| Comments |  |

| Reference  1st author year | Burke et al. 2005 |
| --- | --- |
| Type of study/document | Journal paper |
| Study design | Cross sectional. Comparison of scores and survey of lay panel members. |
| Country | UK |
| Participants/intended audience | 56 lay members. The majority of lay people involved, whilst not medically trained, did have some relevant knowledge or experience: nearly 90% worked in professions related to medical education. |
| Research question/objectives | To evaluate the impact of lay assessors on interview panels for applicants to GP training |
| Research methods | Scores given to interview candidates by lay and medical professional panel members were compared. A survey was sent to lay members. |
| Results/data | The lay assessors were highly motivated to be involved in the selection process, with 49% willing to be involved periodically and a further 47% willing to be involved every time, a twice-yearly commitment. The main motivational factors, identiﬁed in an open question, were that involvement complements their current work and experience (61% respondents), and personal interest in the process (32% respondents). Stated beneﬁts of involvement included an increased understanding of the West Midlands recruitment process (43%), and increased experience in recruitment (34%).  Exploring the marks given to the candidates, overall comparison of the scores revealed no significant difference between marks awarded by the medical and lay assessors (t(206)=0.271, P=0.79). 85% of respondents thought that lay and medical panel members were equally valued.  At present, lay members are mainly recruited through direct contact by the selection organisers, are largely motivated by the relevance of the selection process to their work, and are trained alongside medical assessors. The involvement of wider groups of lay people may require diﬀerent recruitment and training mechanisms.  All assessors attend the same training day, which includes discussion of scoring using videos of role played candidates. Whilst panel members are encouraged to write their marks down separately, there may be within-panel discussion leading to similar views. |
| Main findings | All panel members received the same training. There was no significant difference between lay and medically trained panel member assessments of candidates. |
| Comments |  |

| Reference  1st author year | Dougherty & Easton, 2011 |
| --- | --- |
| Type of study/document | Journal article |
| Study design | A survey of citizen participation in appointed public volunteer boards |
| Country | USA |
| Participants/intended audience | Municipalities in a 10-county area surrounding Pittsburgh, Pennsylvania |
| Research question/objectives | To explore the degree of implementation of citizen participation on Boards and Commissions |
| Research methods | Survey |
| Results/data | 75% of municipalities used appointed volunteer boards as part of their governance structure. The study found that empty seats on volunteer boards were often unfilled. The study found that board members usually received no training or orientation, and that few boards reflected the diversity of local communities. |
| Main findings | Board members received no training. |
| Comments |  |

| Reference  1st author year | Department of Health, 2004 |
| --- | --- |
| Type of study/document | Good practice guidance for the appointment of NHS consultants |
| Study design | Not applicable |
| Country | UK |
| Participants/intended audience | NHS organisations |
| Research question/objectives | Not applicable |
| Research methods | Not applicable |
| Results/data | The core membership of an interview panel should include a lay member (often the chair of the Trust or another non-executive director). All members of the advisory appointments committee should have received appropriate training. This should cover all aspects of the appointments process and focus on equal opportunities and matters which should not be discussed at the interview. It is the responsibility of the employer to ensure that such training has been provided. |
| Main findings | Highlights the importance of training for all panel members in recruitment processes. |
| Comments |  |

| Reference  1st author year | Department of Health, 2006 |
| --- | --- |
| Type of study/document | Guidelines for maternity services liaison committees |
| Study design | Not applicable |
| Country | UK |
| Participants/intended audience | Maternity services |
| Research question/objectives | To provide guidelines in which MSLCs can work effectively and contribute to improving maternity services |
| Research methods | Not applicable |
| Results/data | Members should be committed to working in partnership and to implementing women-centred care. They should be nominated (and replaced) by the group they represent. All new members should receive background and briefing information and meet the chair. Where possible there should be handover between members. User members may need additional information and support when they join, they can be paired with another member to act as a mentor or buddy, a person they can turn to for guidance on how processes and systems work. They may find it useful to have an explanation of the background to items. Joint training of professional and user members has often been considered to be helpful. User members may need additional information about the workings of the services and committee proceedings. A budget for education, training, team building, and induction should be identified. All members should be able to apply to attend training days or conferences.  Members should receive information in a timely fashion, members should have access to a library, internet, the Cochrane Library. They should be offered training in using library resources, and in critical evaluation skills. A budget should be available to reimburse travel, childcare and other out of pocket expenses. The cost to users should be fully recognised including the time commitment. User members can be paid the standard public service loss of earning allowance. The venue needs to be accessible and convenient, the timing needs to be convenient to all members. Provides role specification for user members – duties and person specification.  All members should have a clear brief as to their role on the committee. The following information may be helpful – information on national and local services, and description of the terms of reference of the committee, role of members, previous reports. |
| Main findings | Highlights information, support and practical needs of lay members |
| Comments |  |

| Reference  1st author year | Fletcher & Buggins, 1997 |
| --- | --- |
| Type of study/document | Online summary document (unable to source the full report) |
| Study design | Descriptive |
| Country | UK |
| Participants/intended audience | Public |
| Research question/objectives | To describe the VOICES training programme for parent representatives on Maternity Service Liaison Committees |
| Research methods | Not applicable |
| Results/data | The training sessions are fun, welcoming, informal, friendly, interactive and fun sessions. They encompass introduction of NHS structures and how the healthcare system works, explore the roles and responsibilities of commissioners and managers, identify opportunities for influencing service developments, celebrate successes and aim to equip participants to gather evidence and present a case for change to the committee. |
| Main findings | Comments from participants emphasised that they had gained useful techniques for meetings, confidence to speak up and try to influence |
| Comments |  |

| Reference  1st author year | Frankish, 2002 |
| --- | --- |
| Type of study/document | Journal article |
| Study design | Descriptive overview of the issues |
| Country | Canada |
| Participants/intended audience | Public |
| Research question/objectives | To discuss the challenges associated with citizen participation in regional health boards/councils |
| Research methods | Description only |
| Results/data | The paper reports differing opinions regarding appropriate roles and responsibilities of board members, with discussion regarding the capacity of citizen participants to make health-system decisions. It also reports that there is no explicit set of requisite skills and attributes. It describes board self-assessments as a means of helping boards to explore where improvements in skills or ways of working together are needed. The paper concludes that there is strong evidence that health professionals are the only legitimate and superior decision-makers with citizen participants having less skill or knowledge. |
| Main findings | There may be challenges in establishing roles and responsibilities, and in ensuring equity. |
| Comments |  |

| Reference  1st author year | Gilbert, 2012 |
| --- | --- |
| Type of study/document | Online document |
| Study design | Description |
| Country | UK |
| Participants/intended audience | CCGs |
| Research question/objectives | To assist CCGs in working with lay members and patient representatives |
| Research methods | Not applicable |
| Results/data | While professional development is well-recognised, patient leader development is almost non-existent. Learning should be co-produced with patient leaders. It must focus on what matters to them – for example how to deal with professionals and navigate the systems, therefore building emotional competence and literacy to build trusting relationships, influence decision-making and develop the skills of dialogue. Reports that CLAHRC in NW London ran pilot learning programmes.  Key steps – be clear about the role, purpose and benefits of lay membership and representation. Provide clear guidance on the role, including time commitment, accountability, and identifying conflicts of interest. Clarify the role to the rest of the committee on their status as an equal member, be clear about the voting rationale. Avoid unnecessary terminology, be clear about confidentiality of information and data protection. Financial or other recognition must be agreed in advance, make arrangements for reimbursing travel or covering care. Time constraints of lay members should be considered – timing of meetings, send papers ahead of the meeting, ensure lay members have IT training and support. |
| Main findings | Details information needs of lay members and need to tailor training to member needs/wishes |
| Comments |  |

| Reference  1st author year | Greco et al. 2004 |
| --- | --- |
| Type of study/document | Journal article |
| Study design | Before and after evaluation |
| Country | UK |
| Participants/intended audience | 86 non-executive/lay directors of NHS organisations  Qualities of non-executive directors include possible experience as a carer or users of the NHS. |
| Research question/objectives | To evaluate a one-day training programme to develop confidence, knowledge and skills in appraising clinical governance/quality reports. |
| Research methods | A questionnaire to participants prior to, after the training, and 3 months later.  The training programme firstly consisted of: a two week pre-workshop distance learning pack containing a background and brief to the day plus a case scenario. Delegates were expected to spend around an hour working through the report and accompanying questions. The workshop was a whole day consisting of lectures, small group work, discussion and workshops working through examples. |
| Results/data | There was a significant improvement in delegate knowledge of terminology following the workshop (p=0.001), and this remained three months later. There was a significant increase in skills such as discussing and appraising. There was an impact on confidence elements such as conveying the views of others, asking for clarification, commenting, challenging health professionals when necessary, and helping with surveys/research. There was also an increase in participation elements such as putting ones views across, feeling their advice or ideas are sought, feeling respected, feeling they have contributed. |
| Main findings | The workshop led to improvements in knowledge, attitudes and confidence of the lay directors. |
| Comments | The authors comment that knowledge/skills/attitudes of participants were high prior to the training, with individuals already appearing to be skilled in key areas. |

| Reference  1st author year | Health and Care Professions Council, 2013 |
| --- | --- |
| Type of study/document | Information pack for applicants wishing be a lay member of the council. |
| Study design | Guidance |
| Country | UK |
| Participants/intended audience | Public |
| Research question/objectives | Not applicable |
| Research methods | Not applicable |
| Results/data | The pack provides information about the council and its role, details the aim of recruiting lay members, outlines expectations of the individual and the role, eligibility, and how to apply. It provides a table of core competencies required and indications of evidence that would demonstrate these. It provides information on remuneration, time commitment and location, the appointment and tenure of office if appointed. It documents that “the appropriate training and induction will be provided on appointment”. |
| Main findings | No details of the training are included. |
| Comments |  |

| Reference  1st author year | Healthcare Quality Improvement Partnership, 2013 |
| --- | --- |
| Type of study/document | Report |
| Study design | Guidance |
| Country | UK |
| Participants/intended audience | Healthcare organisations |
| Research question/objectives | To provide guidance on developing patient panels |
| Research methods | Not applicable |
| Results/data | Training and education will be required in the following areas: trust induction as per local induction and volunteer policies, the trust may require an honorary contract to be signed along with health screening and a criminal record bureau check. A local induction could be meeting the team and awareness of local policies. Also consider arranging email account and internet access. Ensure confidentiality agreements are signed. |
| Main findings | Details areas for training. |
| Comments |  |

| Reference  1st author year | Hurtado et al. 2012 |
| --- | --- |
| Type of study/document | Journal article |
| Study design | Descriptive |
| Country | UK |
| Participants/intended audience | Service users with complex needs |
| Research question/objectives | To describe experiences of involving people with learning disabilities and additional needs in the recruitment of staff. |
| Research methods | Description only |
| Results/data | Due to time and other practical constraints such as the large amount of applications, service users were not involved in drawing up the job description, person specification, advertising or shortlisting. They contributed to drawing up a list of their preferred staff qualities, planning the interview day, making a list of questions, deciding on the assessment process, scoring candidates and to the final decision.  As they found service users focused on personal characteristics of candidates, the team used a two-panel system, each focusing on a different aspect of the person specification. The user panel would assess aspects of behaviour and character using a pre-established scoring criteria. The professional panel would assess skills and knowledge. Both panels would join at the end of the day to collaboratively make a decision. The candidate met with the professional panel for 40 minutes, then a shorter interview with service users. The professional panel scoring had a higher weighting however if the user panel had significant concerns a candidate would not be offered the post.  Users received £50 vouchers so that their input was remunerated.  The separate panels were described as successful, it would have been useful to develop user thinking beyond personal qualities, to have a pre-agreed plan in the event of significant disagreement. |
| Main findings | Involvement required careful planning. |
| Comments |  |

| Reference  1st author year | Jenkinson et al. 2014 |
| --- | --- |
| Type of study/document | Journal article |
| Study design | Before and after |
| Country | Australia |
| Participants/intended audience | 55 maternity consumer representatives who had completed a training programme |
| Research question/objectives | What is the effect of a training programme on confidence and engagement for consumer representatives? |
| Research methods | Questionnaire pre and post the programme. The programme consisted of a full day workshop and an information folders. After the workshop participants engaged in mentoring and support activities such as skype, email discussion, online materials. Some people could not continue the training programme due to challenging life circumstances. |
| Results/data | There was a significant increase in perceived confidence (p<0.001), decision-making (p=0.005), confidence in negotiating outcomes and liaising with key people (p=0.029). There was a decline in confidence at 6 months back to baseline levels. |
| Main findings | The programme led to benefits for representatives, although support is needed to maintain confidence levels. |
| Comments |  |

| Reference  1st author year | Jennings & Smith, 2015 |
| --- | --- |
| Type of study/document | Resource manual for lay members of ethical review and similar bodies |
| Study design | Guidance |
| Country | UK |
| Participants/intended audience | Public |
| Research question/objectives | Not applicable |
| Research methods | Not applicable |
| Results/data | The resource includes a definition of terms, an outline of the lay member’s role, a description of the role and functions of the ethical review board and how it is organised, what lay members should expect, how the process will operate and legal requirements as an appendix. |
| Main findings | Highlights the information that might be useful for a lay member including role, the process, expectations and legal requirements. |
| Comments |  |

| Reference  1st author year | Jones & Royse, 2008 |
| --- | --- |
| Type of study/document | Journal letter to Editor |
| Study design | Survey |
| Country | USA |
| Participants/intended audience | 332 citizen review panel members (child protection panels) |
| Research question/objectives | To investigate whether the level of training that panel members had received was associated with perceived effectiveness |
| Research methods | The survey data was examined to evaluate whether reported training was linked to perceived effectiveness of panel membership |
| Results/data | 86% of those who perceived that the citizen’s panel had power to effect change had received training. 80% of those who perceived that the panel was used by the system to pursue its own agenda had received no training. |
| Main findings | The training of panel members is associated with fostering perceptions of effectiveness. |
| Comments | Does not provide any detail of the training. |

| Reference  1st author year | Klitzman, 2012 |
| --- | --- |
| Type of study/document | Journal article |
| Study design | Qualitative interviews with members of university research panel members |
| Country | USA |
| Participants/intended audience | 46 non-affiliated and non-scientific institutional review board members (community members) |
| Research question/objectives | To investigate the role and characteristics of community members |
| Research methods | Interviews with chairs, administrators and members of institutional review boards |
| Results/data | There was confusion regarding who the members were representative of, with the roles appearing to be unclear. They members appeared to have widely varying roles and functions that they were asked to fill. Members were generally appreciated and valued although there was variation in how much the board encouraged the contribution of community members, and there were reports that they could feel intimidated.  Several boards held a detailed orientation to support community members, others did not. A few had a full time staff member to orient and train members. Most community members received little if any orientation, and were told “just do what everyone else is doing”. |
| Main findings | There could be a lack of clarity in role and few received training. |
| Comments |  |

| Reference  1st author year | Lockey et al. 2004 |
| --- | --- |
| Type of study/document | Research report |
| Study design | Qualitative interviews |
| Country | UK |
| Participants/intended audience | 31 interviews with individuals involved in training initiatives for service user involvement, together with visits to training sites |
| Research question/objectives | To investigate what training for involvement in research is available, and what are the views of the training received? |
| Research methods | Initiatives identified and individuals involved with them contacted. |
| Results/data | Training was most valued when it had a clear purpose, and when it was centred around specific tasks or real problems. Participants valued trainers that facilitated the interaction and exchange of ideas. Small group work and role play allowed participants to share experiences. The process of listening to and valuing individual experiences reinforced or redefined that individual experience and understanding was a strength that they brought. Training should be in a safe environment, with time and space to allow contributions. Training was vital to bridge the language gap and make the language as accessible as possible. The issue of payment for undertaking training needs to be considered. |
| Main findings | Training is perceived to be of value and ideally should have a clear purpose, value lay member knowledge, and include understanding of terminology. |
| Comments |  |

| Reference  1st author year | Matka et al. 2010 |
| --- | --- |
| Type of study/document | Journal article |
| Study design | Survey |
| Country | UK |
| Participants/intended audience | Service users and carers and trainees who had been interviewed |
| Research question/objectives | To describe an approach to user involvement in admissions interviews for social work programmes |
| Research methods | A survey was sent out following interviews that had taken place some months before. Service users develop questions for personal suitability interviews at a workshop. The “ideal” qualities of a practitioner are developed. A service user and a clinician interview each applicant. Each interviewer evaluates the candidates’ response using predefined model answers. Interpersonal skills are also assessed. Service users are recruited from local groups and given two afternoons of training by staff, and are then involved in one or two paid half day interview sessions. |
| Results/data | The service users and carers reported that they all gained from being involved. People talked about the gaining of confidence, a feeling of being heard and valued, of gaining knowledge and insight or practical skills. There was a need to understand the rationale for involvement in admissions, how and why. Expectations and responsibilities should be clearly defined, understood and respected by all parties. There were some tensions reported about user capacity to assess applicants, with a need for academics to have final responsibility. Following the evaluation, the Admission Tutor has the final decision. |
| Main findings | Highlights need to consider who makes final decision, and need to be clear about the rationale and include training. |
| Comments |  |

| Reference  1st author year | Monahan & Stewart, 2003 Abstract only available |
| --- | --- |
| Type of study/document | Journal article |
| Study design | Survey |
| Country | Canada |
| Participants/intended audience | 126 scientists and 24 lay panel members who had participated in grant reviews. |
| Research question/objectives | To investigate the attitudes of panel members to the inclusion of non-scientists in the process. |
| Research methods | Questionnaires were sent to all panel members during 1998. Topic included lay member selection, the role of lay panellists, and suggestions for improvement. |
| Results/data | There were significant differences in perceptions of lay member responsibilities between the scientists and lay members (p=0.01), the format of lay grant review (p=0.04), lay panel member contribution to discussion (p=0.01), and understanding of the lay role (p=0.02). |
| Main findings | Professional and lay member perceptions of lay involvement differed. |
| Comments |  |

| Reference  1st author year | Mosconi et al. 2012 |
| --- | --- |
| Type of study/document | Journal article |
| Study design | Survey |
| Country | Italy |
| Participants/intended audience | 99 members of patients’/consumers’ organisations, all represented a voluntary organisation |
| Research question/objectives | To evaluate a training programme in terms of satisfaction of participants and long term impact |
| Research methods | The training course aims to increase critical thinking skills on important issues and health debate and to encourage the autonomy of representatives to be engaged in different forums or committees, and effectively influence decisions. The programme was delivered at a research institute in two cities. The paper describes it as modular with a topic for each module, it is unclear how long the modules were. |
| Results/data | 99% of respondents were satisfied with the programme, were satisfied with the knowledge gained, and most considered that their health knowledge had improved. The majority expressed interest in participating in new editions. 23% changed their role in their organisation after the course. Just over half (59%) reported suggesting changes to policies. There was less satisfaction with more technical modules on “ABC of research, ethics committee, internet in research” most preferred was “uncertainties in medicine”. |
| Main findings | Participants reported a high level of satisfaction with the training, were interested in taking part in further training and reported gains in knowledge. |
| Comments |  |

| Reference  1st author year | NHS England, 2015 |
| --- | --- |
| Type of study/document | Online resource/practical toolkit for the appointment of lay members to CCGs. Drawn up “based on best practice developed by the Appointments Commission” |
| Study design | Guidance |
| Country | UK |
| Participants/intended audience | Guidance for Clinical Commissioning Groups |
| Research question/objectives | Not applicable |
| Research methods | Not applicable |
| Results/data | The resource covers the steps that CCGs may wish to consider in the appointment process for lay members. The information pack that should be provided to potential lay members should include: introduction about the CCG; role and responsibilities; qualities required for the role; details of time commitment, remuneration and induction if appointed; disqualification criteria, and appendices with governance/public life standards. |
| Main findings | The resource provides detail regarding the role and refers to induction, but does not refer to training. |
| Comments |  |

| Reference  1st author year | NHS Wales, 2003 |
| --- | --- |
| Type of study/document | Document outlining training programme for lay members of review panels |
| Study design | Descrotove |
| Country | Wales |
| Participants/intended audience | Potential and existing members of panels |
| Research question/objectives | Not applicable |
| Research methods | The document details that the programme was based on feedback from members and a survey of lay members. |
| Results/data | The programme is completed at home by members. It contains four modules encompassing background information, and the skills and knowledge that are needed to sit as a panel member. The programme directs lay member to other guidance that should be read. New members are encouraged to sit in on a session before undertaking one. The foundation module includes understanding how the NHS works, standards, complaints procedure and the role of lay members. The skills module includes key tasks in the panel member role. |
| Main findings | Description only |
| Comments | A very detailed training course. |

| Reference  1st author year | NHS West Sussex, 2010 |
| --- | --- |
| Type of study/document | Online policy document for the recruitment of lay members to groups or committees |
| Study design | Written policy |
| Country | UK |
| Participants/intended audience | CCGs |
| Research question/objectives | Not applicable |
| Research methods | Not applicable |
| Results/data | It is good practice to have at least two lay members on a committee. This maximises the opportunity to provide each other with peer support and increases likelihood that at least one will attend. Lay members are not expected to represent the views of the wider community (unless recruited because they are a member of a specific group and are authorised to speak on behalf of that group).  The document includes: all lay members will be required to sign and abide by a confidentiality agreement, and will be required to have a criminal records bureau check if they will have direct contact with potentially vulnerable individuals. Lay members should not sit on more than three CCG groups or committees to ensure opportunities for as many different people as possible.  The requirement for specific knowledge, experience or expertise should be made clear in a Lay Member Role Description.  Newly recruited members are provided with a Lay Member Information Pack. Once lay members are appointed a public engagement team is responsible for ongoing support, information and training. Lay members should be given the contact details of an identified member of staff in the department so they know who to contact for support and advice.  Lay members must be provided with enough support, information and training as they need to fulfil their role and participate fully. New members should be invited to a short orientation meeting to provide information and discuss their support and training needs.  Meeting papers should be provided in good time by the method chosen by the lay member. If meeting papers are numerous, they should be sent hard copies or printing costs should be reimbursed.  Full details of venues including postcode, a location map, directions and information on parking should be provided. Use plain English at all times, if the use of jargon and abbreviations is necessary ensure they are fully explained. Consider devising a Glossary of common terms for the benefit of lay members.  The role description should include contact details for the organiser, the name of the activity, the remit, the role that the participant will fulfil, any particular skills or knowledge required, the format of the activity, the time required the period, and the induction and support that will be provided. |
| Main findings | The document emphasises the need for clarity regarding the role and need for orientation and support. |
| Comments |  |

| Reference  1st author year | O’Connor et al. 2007 |
| --- | --- |
| Type of study/document | Journal article |
| Study design | Survey, focus groups and interviews |
| Country | Ireland |
| Participants/intended audience | Service user members of review teams, staff, representatives of organisations |
| Research question/objectives | To evaluate the role and participation of service user members in accreditation assessments of healthcare organisations. |
| Research methods | Views were sought after seven reviews to accredit healthcare organisations had been completed that had included service users on the review panel. Training was provided to service user members on the process and skills that would be required. |
| Results/data | Benefits of user involvement were reported to be adding credibility and balance to the process. Good communication and interpersonal skills, excellent powers of observation and objectivity were identified as important traits for service users.87% of staff agreed or strongly agreed that users possessed the skills/characteristics required. 100% of service user members strongly agreed that self-assessment against core competency criteria was particularly beneficial. 100% agreed that the training they had received was beneficial and that it gave them a good insight into their role. The half day training was considered to be too short. This since has been changed to 2 days. They felt training could be improved by introducing a shadowing component and focusing more on report writing. There were a number of issues in relation to the role of the user assessors. Some were asked to contribute to the rating of the organisation and others were not, depending on who the team leader was. This has now been clarified and staff facilitate the contribution of users to rating. Some provided only a verbal input to the report, others a written input. All participants reported that there was a role for user members in feeding back/debriefing. This has since been changed with a template for written reports or the option of presenting feedback verbally offered. |
| Main findings | Having core skills/knowledge criteria was useful, an adequate period of training was important, the role of users needed clarifying. |
| Comments |  |

| Reference  1st author year | O’Hara et al. 2015 |
| --- | --- |
| Type of study/document | Report |
| Study design | Survey |
| Country | Canada |
| Participants/intended audience | Lay representatives on health research committees |
| Research question/objectives | What is the role of lay representatives on health research committees |
| Research methods | Online survey |
| Results/data | There was a high degree of agreement between the way non-lay representatives saw their lay colleagues and the way lay representatives see themselves. The term “community representative” or “community member” was the preferred. The question of what community does the representative serve is important. Almost all respondents indicated that education is a large part of the solution to having a well-informed and productive lay representative. The education needed to be ongoing, and training coming from multiple sources – online, face to face mentoring, workshops and other forms of instruction.  The volume of material and associated time commitment was raised as an issue, with the suggestion that it could be beneficial to have two lay representatives to reduce the burden.  Some respondents reported that they had sufficient expertise and as such did not receive additional training, several reported that they received little or no training or were self-taught. Training was generally – orientation sessions, training sessions, reading materials or online tutorials. Need for training to empower public to understand role, science education/clarification of medical terminology, act as an observer before joining committee, to clarify committee processes mission values strategic priority, for role clarification.  Around half were reimbursed in the form of a payment, meal, travel, conference attendance or general expenses. |
| Main findings | Time commitment and training needs were highlighted. |
| Comments |  |

| Reference  1st author year | Oliver et al. 2001 |
| --- | --- |
| Type of study/document | Journal article |
| Study design | Action research including desk-based research and interviews |
| Country | UK |
| Participants/intended audience | Organisations involved in the NIHR Health Technology Assessment programme, researchers, consumers from self-help and patient groups. |
| Research question/objectives | What are the processes for enabling consumer involvement in the research programme, and what are consumer experiences of involvement in research funding panels. |
| Research methods | A broad range of views were sought from staff, consumers, panel members of the HTA programmes via interview, post or email, all panel members were invited to a whole day meeting. |
| Results/data | Learning from others was a recurring theme, new panel members learnt from more experienced colleagues. New members appreciated an induction day but found this only a beginning to a long apprenticeship and would appreciate on-going support perhaps through a system of mentorship. Support suggested for new members of panels was: practice sessions; someone to phone when something was unclear; opportunities to meet others.  Language was repeatedly mentioned as a barrier to involvement. Specialist jargon could be alienating but business may be impeded if everything was to be “translated” into non-specialist language. Consumers suggested that they would benefit from a glossary or medical dictionary, and being able to telephone someone for an explanation if they did not understand.  Management and committee terminology were also raised as perplexing consumers – executive, speaking through the chair, trajectory, secondary publications etc. Confusions were not always predictable and there was a need to check constantly that discussion was understood. The study suggested training consumers to participate in professional debates, and training professionals to listen to consumers in their own terms. |
| Main findings | Challenges to involving users were cultural divides, language barriers, and a need for skill development among consumers and others. |
| Comments |  |

| Reference  1st author year | Oxfordshire, 2010 |
| --- | --- |
| Type of study/document | Induction booklet |
| Study design | Policy document |
| Country | UK |
| Participants/intended audience | Newly recruited lay members of Safeguarding Children Board |
| Research question/objectives | N/A |
| Research methods | N/A |
| Results/data | Comprises sections on: Introduction; Induction process; Meeting schedule; Contact details; Historical context; Local context; Role of Local Safeguarding Children Board; Role of Lay Members; How lay members fulfil their role; Conclusion; Jargon buster; Self Assessment Checklist (not included in pack). It is described as a self evaluation framework to assist lay members in satisfying themselves that they have all the information, tools and assistance to help them fulfil their role on the board.  There are references for suggested extra reading.  Induction process includes a buddy system. The buddy is expected to meet to discuss use of the booklet. It is suggested that the buddy meets immediately prior to the first two board meetings. A phone call also should take place following the board meeting to reflect and discuss any issues. There is a formal meeting with the Chair of the Board after 6 months. |
| Main findings | N/A |
| Comments |  |

| Reference  1st author year | Parkes et al. 2014 |
| --- | --- |
| Type of study/document | Journal article |
| Study design | Survey evaluation |
| Country | UK |
| Participants/intended audience | Patient groups |
| Research question/objectives | To describe and evaluate a research awareness training programme for public involvement in research (Public Engagement in Research Programme). |
| Research methods | The programme was developed and trialled with a number of different patient groups including learning disability service users, women in a secure mental health hospital, users of a child and adolescent mental health service. The format of the workshops varied from 2 hours once a week for 8 weeks to 2 weekends. |
| Results/data | Limited evaluation data. Comments on a post-course evaluation questionnaire indicated that participants valued the course, they particularly enjoyed working with others, findings things a challenge, teamwork. Core elements of the training model identified by the study are: collective teamwork; context of PPI involvement; external support; self-reflection; research methods. |
| Main findings | Limited relevance to review, emphasises importance of training PPI members for involvement. |
| Comments |  |

| Reference  1st author year | Pickard et al. 2001 |
| --- | --- |
| Type of study/document | Journal article |
| Study design | Survey |
| Country | UK |
| Participants/intended audience | Lay members of primary care group boards |
| Research question/objectives | What has been the level of involvement of lay members in decision-making and the engagement of the public? |
| Research methods | Analyses data from the lay element of the annual Tracker survey of PCGs in England. |
| Results/data | The role of lay members was ill-defined and as a result was variable between organisations, involvement in decision-making tended to be low. There was some ambiguity around who the lay member believed themselves to be representing, and their role tended to be unclear. Their role was described as being hampered due to a lack of specialist knowledge. Training was sporadic and varied considerably and ranged from a one day seminar and induction to specific training in aspects of public participation. Lay members highlighted that assimilation of a huge amount of information may be required and this needed time to absorb and build up knowledge. |
| Main findings | A lack of role clarity hampered influence, there is a need for time to get to grips with large amounts of information. |
| Comments |  |

| Reference  1st author year | Richardson et al. 2013 |
| --- | --- |
| Type of study/document | Journal article |
| Study design | Descriptive |
| Country | UK |
| Participants/intended audience | Lay members of interview panels for students applying to operating department practitioner programmes |
| Research question/objectives | To describe a lay member interviewer initiative |
| Research methods | Describes the setting up and involvement of lay members in prospective healthcare student interviews over a 3 year period. |
| Results/data | The university department already had an established strategy for service user involvement, including a payment policy (payment is offered for time, travel expenses and child care or personal assistant costs), a part-time coordinator and a database of service users who were willing to participate. Initial considerations among the ODP team concerned the need to ensure that service users would be aware of limitations, such as time and focus, have a clear understanding of the rules of confidentiality, be reasonably confident in front of applicants, the experience would be positive for candidates, and what would happen if service users were in disagreement with the choice of an applicant’s selection?  The preparation or induction consisted of: information about the profession; information about the course; the criteria for applying for the course; documentation; the interview process; what the service user’s role would be within that process; the scoring process; issues of confidentiality, equality, diversity and data protection. Academic staff also discussed some typical interview situations that they had encountered.  This induction process was important in order to build relationships with the service users, and for the service users to highlight any concerns or identify special requirements.  The department has a pool of 7 service users to draw upon. Each interview panel consists of an academic, a practitioner and a service user. Service users are matched with academics and practitioners to allow for different levels of experience.  Questions for the candidates are divided equally between panel members. Service users provided interesting and useful reflections on the wording of some of the interview questions. User members are often the members of the panel who ask questions about typical scenarios (developed by user members). This provides the opportunity to observe how candidates respond to, and communicate with, service users.  After each interview, members discuss their scores using the interview profile. Differences in scores between panel members are discussed until a consensus is reached. The chair of the panel (the academic) records the final score for each candidate. At the end of the round of interviews each year, a meeting is held to review all the candidates and to decide which applicants will receive an offer. Service users are invited to this meeting, so that they can see how the process is completed.  An evaluation form is completed by all interviewers at the end of each round of interviews. The feedback is discussed before the new round of interviews at a meeting where all interviewers are invited. |
| Main findings | The involvement required time, resources and effort, but was perceived to be outweighed by the benefits.  Practical problems - some interviewers, especially those in rural areas, were unable to make the journey to the university. A standby system was put into place with 24 hours’ notice. Some of the rooms the interviews were held in were not sufficiently heated for less mobile service users.  Advantages described were in regard to service users offering a different perspective, greater understanding of service user priorities. Users gave added value to the final score and confidence to the interview teams that they are selecting the ‘right’ candidates. Service users felt that their contributions were valued, students also were positive. Fears around disagreement were not realised, there was often agreement between panel members and, where scoring differed significantly, agreement was achieved through further discussion. |
| Comments |  |

| Reference  1st author year | Roberts et al. 2010 |
| --- | --- |
| Type of study/document | Journal article |
| Study design | Interviews and survey |
| Country | UK |
| Participants/intended audience | Project team, staff, lay assistants, interviewees. |
| Research question/objectives | To describe and evaluate the inclusion of lay people in student nurse interviews |
| Research methods | The paper describes the development and introduction of the initiative. Focus groups for assistants, and for students were used to inform the process of involvement and to develop materials to be used. It provides limited evaluation data in the form of quotes from those involved and some quotes from interviewees who completed a postal survey. |
| Results/data | The project team developed a set of selection criteria and processes to recruit a pool of lay people. The HR department were included to ensure that selection processes and issues of diversity were acknowledged. An advert, role description and person specification were developed. Selection criteria included enhanced CRB clearance and completion of mandatory NHS Trust volunteering training; education to at least GCSE level, at least 6 months experience as a volunteer. The term “lay assistant” was used. The assistants were employed on casual contracts and were given an initial briefing session – introduction to the school, introduction to the project, a tour of the facilities, a chance to contribute ideas and ask questions. There was an informal buffet for everyone to meet.  The materials developed and used were firstly, a briefing sheet for the assistants setting out the steps in the process including timings. Also, a sheet providing examples of question types that were to be used in the interviews, together with a feedback form on which assistants scored applicants and gave comments.  A training workshop was provided which included: awareness of good practice in interviewing; awareness of equality and diversity issues and confidentiality; using documentation; feeding back to the interview panel, clarification on boundaries of role, an opportunity to see the rooms that would be used and talk to administrators, role play scenarios.  Lay assistants met the applicants separately for half an hour before the interview and did not take part in the interview. They were given tasks to do with the candidates around personal qualities and specific questions. The lay assessment did not affect the outcome of the interview in this project but this is intended to be changed for the final implementation.  The lay assistants rated the experience positively and had found the materials helpful. The project team highlighted the importance of good preparation, and reported that the interview process was more relaxed and a better experience for the candidate. The authors recommended having strategies in place to deal with differences of judgement, a sufficient number of lay individuals needed to be available, sufficient training should be planned, lay people should be briefed fully and committed to being involved in the process for a complete cycle, initial and ongoing training should be provided. Resources need to be allocated (there are no details of payment in the paper). |
| Main findings | The paper details training provided and an alternative way of public assistants being involved in an interview. |
| Comments |  |

| Reference  1st author year | Ukpong et al. 2012 |
| --- | --- |
| Type of study/document | Conference presentation |
| Study design | Controlled Before and after, interviews |
| Country | Nigeria |
| Participants/intended audience | Researchers |
| Research question/objectives | To describe a five year project which aimed to increase the role of laypersons on ethics committees |
| Research methods | The training focused on three curricula: how to review a research proposal and provide constructive feedback; how to monitor research; how to evaluate for community engagement in research protocols. Each curriculum was delivered over 1.5-5 days. A self-training manual was also developed. |
| Results/data | Lay people who had been trained improved their skills and knowledge, were better able to monitor researchers and were better able to discuss research monitoring. There was no significant difference in knowledge between those trained and untrained which the researchers attributed to training “rubbing off” on those untrained. Comparison of an ethics committee which had trained members with those who had no trained members indicated that the one with trained members had greater knowledge (p=0.002). |
| Main findings | The study recommended that lay members of ethics committees should take update training at least once a year. |
| Comments |  |

| Reference  1st author year | University of Keele, 2015 |
| --- | --- |
| Type of study/document | Online document |
| Study design | Guidance |
| Country | UK |
| Participants/intended audience | Public (potential lay interviewers) |
| Research question/objectives | Information for applicants to be lay interviewers |
| Research methods | Not applicable |
| Results/data | The document outlines the time commitment, whether there will be payment, the location, what their contribution would be to the process, and their role, what may be gained, what skills/qualities are needed. It outlines that interviewers will receive mandatory equality and diversity training, and introduction to the University School “you will be fully prepared and supported in your role”. It provides a list of attributes in the form of a “skills profile”. |
| Main findings | .Provides clear guidance to prospective lay interviewers. |
| Comments |  |

| Reference  1st author year | Wandsworth CCG, 2013 |
| --- | --- |
| Type of study/document | Online document |
| Study design | Policy document/guidance |
| Country | UK |
| Participants/intended audience | Local services |
| Research question/objectives | Provides Top Tips on appointing patient representatives |
| Research methods | Not applicable |
| Results/data | There is a need to be clear what you are expecting and what the boundaries are. A role or job description is needed which should set out key tasks that will be required. Representatives will need help in understanding the structure and working relationships of the NHS, arrange some information or training on this. There should be a dedicated member of staff to whom they can turn to for support. It is important to stress any confidentiality issues, a confidentiality policy is available. |
| Main findings | Highlights a need for clarity in role and training in confidentiality/processes. |
| Comments |  |
